# Author Correction: Isospin competitions and valley polarized correlated insulators in twisted double bilayer graphene

**DOI:** 10.1038/s41467-025-56809-7

**Published:** 2025-02-12

**Authors:** Le Liu, Shihao Zhang, Yanbang Chu, Cheng Shen, Yuan Huang, Yalong Yuan, Jinpeng Tian, Jian Tang, Yiru Ji, Rong Yang, Kenji Watanabe, Takashi Taniguchi, Dongxia Shi, Jianpeng Liu, Wei Yang, Guangyu Zhang

**Affiliations:** 1 Beijing National Laboratory for Condensed Matter Physics and Institute of Physics, Chinese Academy of Sciences, Beijing, 100190 China; 2 School of Physical Sciences, University of Chinese Academy of Sciences, Beijing, 100190 China; 3 School of Physical Sciences and Technology, ShanghaiTech University, Shanghai, 200031 China; 4 Advanced Research Institute of Multidisciplinary Science, Beijing Institute of Technology, Beijing, 100081 China; 5 Songshan Lake Materials Laboratory, Dongguan, 523808 China; 6 Research Center for Functional Materials, National Institute for Materials Science, 1-1 Namiki, Tsukuba, 305-0044 Japan; 7 International Center for Materials Nanoarchitectonics, National Institute for Materials Science, 1-1 Namiki, Tsukuba, 305-0044 Japan; 8 ShanghaiTech Laboratory for Topological Physics, ShanghaiTech University, Shanghai, 200031 China

Correction to: *Nature Communications* 10.1038/s41467-022-30998-x, published online 07 June 2022

In the version of the article initially published, the National Natural Science Foundation of China grant no. 12074413 was written incorrectly and has now been amended in the HTML and PDF versions of the article.

