# Supplementary material for: Isospin competitions and valley polarized correlated insulators in twisted double bilayer graphene
Source: Nat Commun. 2022 Jun 7;13:3292. doi: 10.1038/s41467-022-30998-x (PMC9174269; doi:10.1038/s41467-022-30998-x)
Supplement: Supplementary file 1 — Supplementary Information [file 41467_2022_30998_MOESM1_ESM.pdf]

Supplementary information for "Isospin competitions and valley polarized correlated insulators in twisted double bilayer graphene"

Le Liu[1,2], Shihao Zhang[3], Yanbang Chu[1,2], Cheng Shen[1,2], Yuan Huang[4], Yalong Yuan[1,2], Jinpeng Tian[1,2], Jian Tang[1,2], Yiru Ji[1,2], Rong Yang[1,5], Kenji Watanabe[6], Takashi Taniguchi[7], Dongxia Shi[1,2,5], Jianpeng Liu[3,8], Wei Yang[1,2,5*] & Guangyu Zhang[1,2,5*]

[1] *Beijing National Laboratory for Condensed Matter Physics and Institute of Physics, Chinese Academy of Sciences, Beijing 100190, China*
[2] *School of Physical Sciences, University of Chinese Academy of Sciences, Beijing, 100190, China*
[3] *School of Physical Sciences and Technology, ShanghaiTech University, Shanghai 200031, China*
[4] *Advanced Research Institute of Multidisciplinary Science, Beijing Institute of Technology, Beijing, 100081, China*
[5] *Songshan Lake Materials Laboratory, Dongguan 523808, China*
[6] *Research Center for Functional Materials, National Institute for Materials Science, 1-1 Namiki, Tsukuba 305-0044, Japan*
[7] *International Center for Materials Nanoarchitectonics, National Institute for Materials Science, 1-1 Namiki, Tsukuba 305-0044, Japan*
[8] *ShanghaiTech Laboratory for Topological Physics, ShanghaiTech University, Shanghai 200031, China*

* Corresponding authors. Email: wei.yang@iphy.ac.cn; gyzhang@iphy.ac.cn

Supplementary Note 1: Theoretical calculations.

1.1. **Continuum Hamiltonian for twisted double bilayer graphene.**

First, we introduce the low-energy effective Hamiltonian of the twisted double bilayer graphene (TDBG) of the K valley which is expressed as

$$H_\mu^0 = \begin{pmatrix} H_\mu^\alpha & \mathbb{U}_\mu \\ \mathbb{U}_\mu^\dagger & H_\mu^{\alpha'} \end{pmatrix}, \tag{1}$$

in which H_μ^α and $H_\mu^{\alpha'}$ are the low-energy effective Hamiltonians for the top bilayer and bottom bilayer graphene with chiral stackings α and α', and $\mu = \pm$ denotes the K'/K valley. Here,

$$H_\mu^\alpha = \begin{pmatrix} h_\mu^0(\boldsymbol{k}) & h_\alpha \\ h_\alpha^\dagger & h_\mu^0(\boldsymbol{k}) \end{pmatrix}, \tag{2}$$

where $h_\mu^0(\boldsymbol{k}) = -\hbar v_F(\boldsymbol{k}-\boldsymbol{K})\cdot\sigma_\mu$ is the low-energy effective Hamiltonian for monolayer graphene near the Dirac point **K**, and $\sigma_\mu = (\mu\sigma_x, \sigma_y)$. h_α is defined as the interlayer hopping matrix of the chiral multilayer graphene with stacking chirality $\alpha = \pm$ representing AB or BA stacking[1-3], with

$$h_+ = \begin{pmatrix} t_2 f(\boldsymbol{k}) & t_2 f^*(\boldsymbol{k}) \\ t_\perp - 3t_3 & t_2 f(\boldsymbol{k}) \end{pmatrix}, \tag{3}$$

where t_2 =0.21eV, t_3 ≈0.05eV, and $t_\perp$ =0.48eV are extracted from the Slater-Koster hopping parameters in Ref. 4[4]. In this matrix, we use the phase factor $f(\boldsymbol{k}) = e^{-i\sqrt{3}ak_y/3} + e^{i(k_x a/2+\sqrt{3}ak_y/6)} + e^{i(-k_x a/2+\sqrt{3}ak_y/6)}$. The interlayer hopping with - stacking chirality $h_- = h_+^\dagger$.

The off-diagonal term U represents the coupling between the twisted double bilayers,

$$\mathbb{U}_\mu = \begin{pmatrix} 0 & 0 \\ U_\mu(\boldsymbol{r})e^{i\mu\Delta\boldsymbol{K}\cdot\boldsymbol{r}} & 0 \end{pmatrix}, \tag{4}$$

in which the matrix U describes the tunneling between the Dirac states of the twisted bilayers neat the twist interface[5, 6]

$$U_\mu(\boldsymbol{r}) = \begin{pmatrix} u_0 g_\mu(\boldsymbol{r}) & u_0' g_\mu(\boldsymbol{r} - \boldsymbol{r}_{AB}) \\ u_0' g_\mu(\boldsymbol{r} + \mu\boldsymbol{r}_{AB}) & u_0 g_\mu(\boldsymbol{r}) \end{pmatrix}, \tag{5}$$

where $\boldsymbol{r}_{AB} = (\sqrt{3}L_s/3, 0)$, u_0' and u_0 denote the intersublattice and intrasublattice interlayer tunnelling amplitudes, with u_0'≈0.098eV, and u_0≈0.078eV[6]. u_0 is smaller than u_0' due to the effect of atomic corrugations[6, 7]. $\Delta\boldsymbol{K} = \boldsymbol{K}' - \boldsymbol{K} = (0, 4\pi/3L_s)$ is the shift between the Dirac points of the double bilayers. The phase factor in this tunnelling matrix $g(\boldsymbol{r})$ is defined as $g_\mu(\boldsymbol{r}) = \sum_{j=1}^{3} e^{-i\mu\boldsymbol{q}_j\cdot\boldsymbol{r}}$, with $\boldsymbol{q}_1 = (0, 4\pi/3L_s)$, $\boldsymbol{q}_2 = (-2\pi/\sqrt{3}L_s, -2\pi/3L_s)$, and $\boldsymbol{q}_3 = (2\pi/\sqrt{3}L_s, -2\pi/3L_s)$.

If we impose an out-of-place displacement field *D* on the TDBG system, the displacement field will introduce an on-site energy to the lth layer (l = 1, 2, 3, 4) as $U_l = (l-1)U_d/3$, in which $U_d = -eDd/\varepsilon_{eff}$, d=10.05Å represents the total thickness of the TDBG system, and ε_{eff}=10 refers to the effective dielectric constant of the twisted double bilayer graphene system. Thus, the displacement field *D* = 0.4 V/nm refers to U_d = 0.04eV in the continuum model.

The energy bands of TDBG with twist angle 1.21° and 1.38° at different electric displacement fields are shown in the Supplementary Fig. 1 and 2, respectively. The Chern number of highest valence band (VB) and lowest conduction band (CB) of K valley are both *C*=1 under U_d =0.04 eV displacement field. If the vertical displacement field is enhanced to the U_d=0.07 (0.09) eV, the highest VB will intersect with remote VB, while the lowest CB is still isolated. In this situation the Chern number of the lowest CB is *C*=1, but the Chern number of the highest VB can't be calculated. We note that under this displacement field, the extremely flat band of the highest VB along $\Gamma_s - \mathrm{M}_s$ direction is exactly located at the *ν*=-2 which contributes to the van Hove singularity as shown in the Supplementary Fig. 1(c).

1.2. **The coulomb interactions in the twisted graphene system.**

In this work, we consider the inter-site Coulomb interactions

$$H_c = \frac{1}{2N_s}\sum_{\alpha\alpha'}\sum_{\boldsymbol{k}\boldsymbol{k}'\boldsymbol{q}}\sum_{\sigma\sigma'} V(\boldsymbol{q})\hat{c}^\dagger_{\boldsymbol{k}+\boldsymbol{q},\alpha\sigma}\hat{c}^\dagger_{\boldsymbol{k}'-\boldsymbol{q},\alpha'\sigma'}\hat{c}_{\boldsymbol{k}',\alpha'\sigma'}\hat{c}_{\boldsymbol{k},\alpha\sigma} \tag{6}$$

where α stands for the layer and sublattice indices, **k** and **q** are atomic wavevectors, and σ refers to the spin index. Here we define the screened Coulomb interaction $V(q)$ as $V(q) = e^2/(2\Omega_M \epsilon\epsilon_0\sqrt{q^2+\kappa^2})$, in which Ω_M means the area of moiré supercell, κ is the inverse screening length 0.005Å^{-1} and ϵ=10 denotes background dielectric constant. In the moiré system with small twist angle, the Coulomb interactions can be further decomposed into the intravalley one and the intervalley one, with the former being two orders of magnitudes larger than the latter, thus we only consider the intravalley part of the inter-site Coulomb interaction.

In addition to the inter-site Coulomb interactions, on-site Hubbard interaction also contributes to the emergence of correlated states at half filling where the on-site Hubbard interaction is expresses as

$$H_{on-site} = \frac{U_0 a^2}{N_M L_s^2} \sum_{\boldsymbol{kk'q}} \sum_{\alpha} \hat{c}^\dagger_{\boldsymbol{k+q},\alpha\uparrow} \hat{c}^\dagger_{\boldsymbol{k'-q},\alpha'\downarrow} \hat{c}_{\boldsymbol{k'},\alpha'\downarrow} \hat{c}_{\boldsymbol{k},\alpha\uparrow} \;, \qquad (7)$$

where $a\,(L_s)$ denotes the atomic (moiré) lattice constant, and U_0~1-5eV represents the atomic Hubbard U value in twisted graphene systems.

We make the Hartree-Fock (HF) approximation about the inter-site Coulomb interactions and on-site Hubbard interaction, and project these interactions onto a few energy bands around the charge-neutrality point for each valley each spin. We start with 32 possible initial trial wavefunctions in the valley-spin-sublattice space, and perform the self-consistent calculations to solve the interacting Hamiltonian.

When the external vertical magnetic field is imposed on the TDBG system, the magnetic field will bring the spin Zeeman effect and orbital Zeeman effect which can be conveniently described using the orbital g factor defined in the subspace of the six bands near the CNP[8-12]. We define the matrix element of the orbital g factor in the flat band basis as

$$\hat{g}^{\mu}_{mm'}(\widetilde{\boldsymbol{k}}) = \frac{-im_e}{2\hbar^2} \sum_{l} \left(\frac{1}{E^{\mu}_{m\widetilde{\boldsymbol{k}}} - E^{\mu}_{l\widetilde{\boldsymbol{k}}}} + \frac{1}{E^{\mu}_{m'\widetilde{\boldsymbol{k}}} - E^{\mu}_{l\widetilde{\boldsymbol{k}}}} \right) \left(\hat{v}^{x,\mu}_{ml} \hat{v}^{y,\mu}_{lm'} - \hat{v}^{y,\mu}_{ml} \hat{v}^{x,\mu}_{lm'} \right), \qquad (8)$$

in which m,m' refer to the band indices, and l refers to other band index, $E^{\mu}_{m\widetilde{\boldsymbol{k}}}$ represents the non-interacting energy of valley μ at moiré wavevector $\widetilde{\boldsymbol{k}}$, and $v^{a,\mu} = \partial H(\boldsymbol{k})/(\hbar\partial k_a)$ $(a = x, y)$ stands for the velocity operator for valley μ. With the orbital g factor, the orbital magnetic Zeeman effects can be expressed as $((H^{\mu}_{Zeeman})_{mm'}(\widetilde{\boldsymbol{k}}) = \mu_B \hat{g}^{\mu}_{mm'}(\widetilde{\boldsymbol{k}}) B_z$ which B_z is the z component of the external magnetic field.

We present the reciprocal space distribution of orbital g factor of CB from K valley under U_d=0.09 eV displacement field in the Supplementary Fig. 3(a). The Landau level spacing $\hbar\omega_c = e\hbar B/m^*$ is only 92 μeV which is much smaller than orbital Zeeman splitting ~0.3-0.58 meV. Thus, in the following calculations with magnetic field, it is reasonable to neglect Landau-level quantization effect under weak magnetic field.

We focus on the HF ground state at ν=2 as shown in the Supplementary Fig. 3. Without external

magnetic field and on-site Hubbard interactions, the calculated ground states are two degenerate states[3]: one is valley polarized (VP) state, and the other is a spin polarized (SP) state. But if we introduce the on-site Hubbard interaction, the SP state will become the unique ground state. For example, when on-site Hubbard energy U_0 is set as 0.5 eV, the total energy of VP state will be 0.256meV higher than that of SP state. When the vertical magnetic field is enhanced, the orbital Zeeman effect leads to the valley polarization and drives doped system into the VP insulator with total Chern number $|C|$=2 where there are two unoccupied bands from same valley.

As for the HF ground state at v=-2, the system prefers to the valley polarization driven by orbital Zeeman effect and spin polarization disappears when the vertical magnetic field is increased to 2T under U_d=0.07 eV (Supplementary Fig. 4). But different from fully valley polarization ($\langle\tau_z\rangle$=±2 means two occupied bands belongs to same valley), the valley polarization at this phase is only $\langle\tau_z\rangle$=-1.39 which is somewhat cancelled by finite intervalley coherent (IVC) order. Thus, the ground state at this time is valley polarized mixed with IVC order, and the global gap opens with the assistance of IVC order. By the way, the IVC order makes the correlated insulator at v=-2 become a topological trivial insulator, which is consistent with our experiment results.

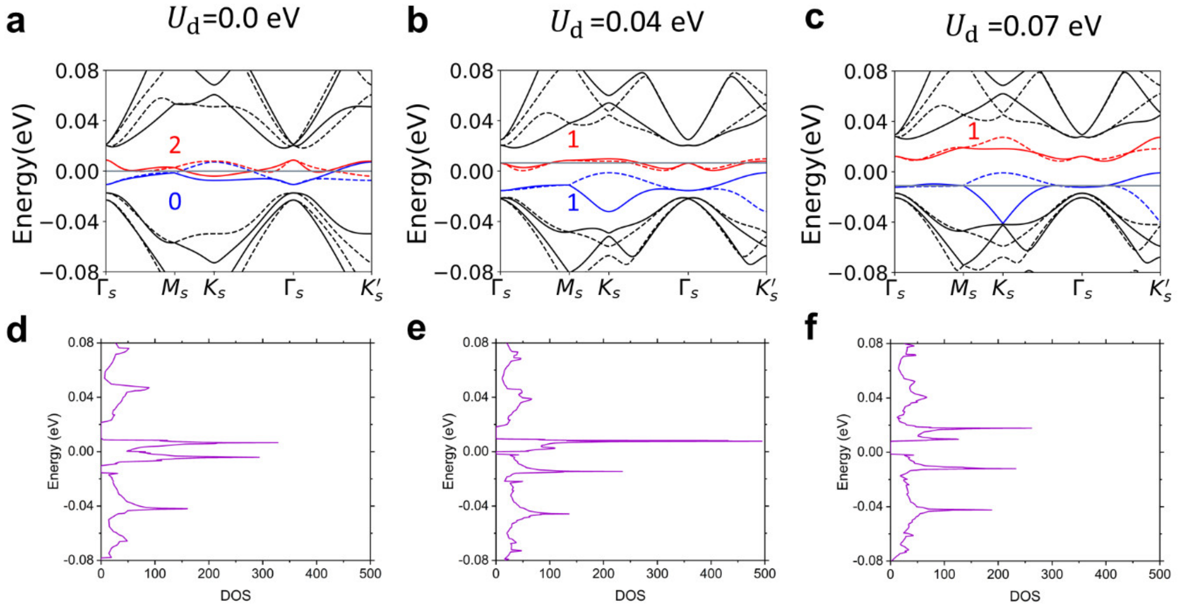

Supplementary Figure 1. **The energy bands and density of states of twisted AB-BA double bilayer graphene with twist angle θ = 1.21°**. U_d = 0 eV (**a**), (**d**), U_d = 0.04 eV (**b**), (**e**) and U_d = 0.07 eV (**c**), (**f**). The energy bands from K (K') valley are present with solid (dashed) lines. The Fermi levels of v = 0 (**a**), v = 2 (**b**) and v = -2 (**c**) are remarked with grey line.

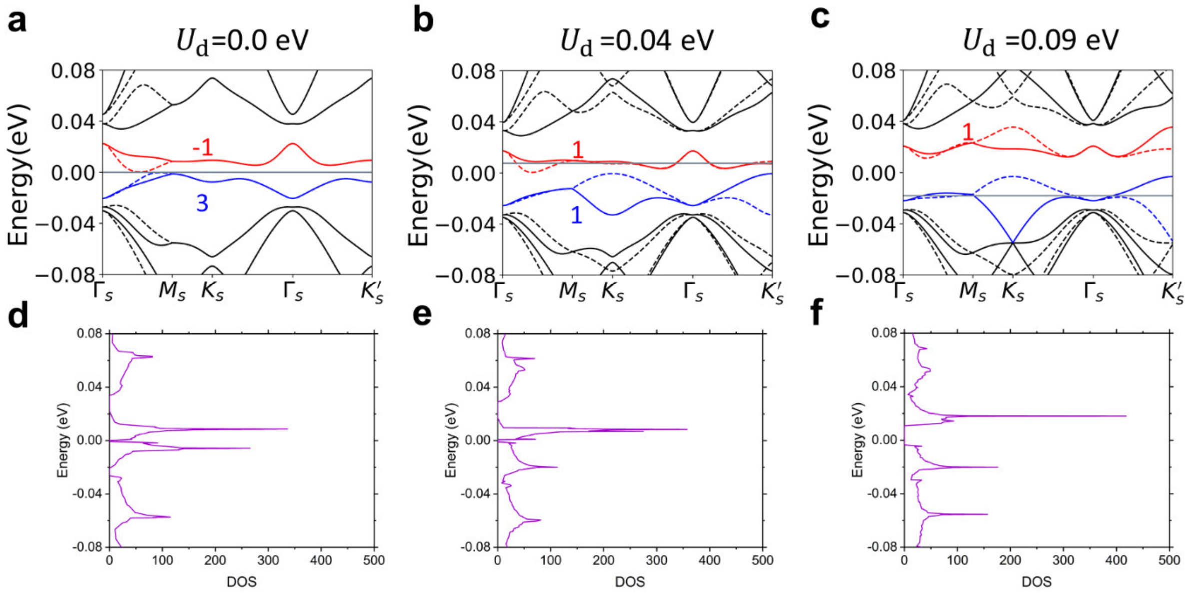

Supplementary Figure 2. **The energy bands and density of states of twisted AB-BA double bilayer graphene with twist angle θ=1.38°**. U_d=0 eV (**a**), (**d**), U_d=0.04 eV (**b**), (**e**) and U_d=0.09 eV (**c**), (**f**). The energy bands from K (K') valley are present with solid (dashed) lines. The Fermi levels of v=0 (**a**), v=2 (**b**) and v=-2 (**c**) are remarked with grey line.

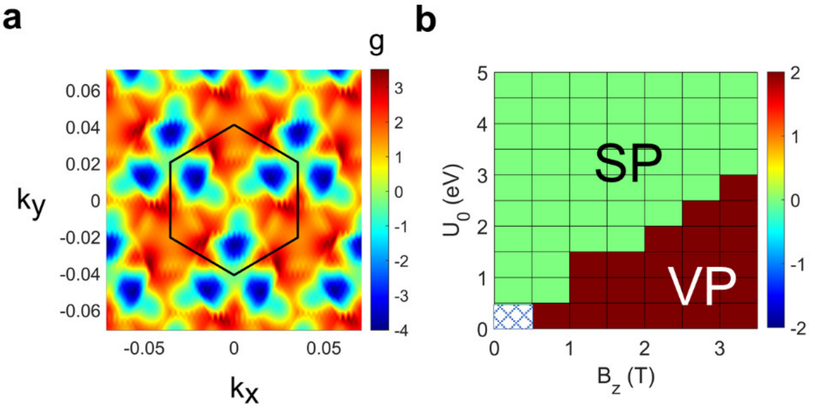

Supplementary Figure 3. **Spin and valley polarized state at** ν**=2 with twisted angle** θ**=1.38°**. **a**, The distribution of orbital g factor in the reciprocal space at $\nu = 2$ under U_d = 0.09 eV displacement field. **b**, The Hartree-Fock phase diagram in the parameter space of on-site Hubbard interaction U_0 and out-of-plane magnetic fields B_z at $\nu = 2$ under U_d = 0.09 eV displacement field. The shaded block means that the spin polarized (SP) and valley polarized (VP) states are degenerate at the origin.

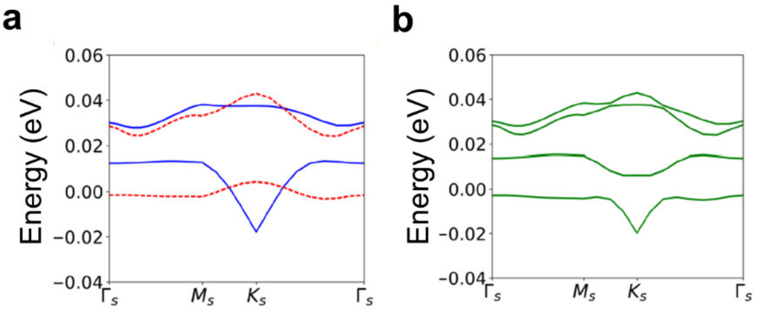

Supplementary Figure 4. **The HF energy bands of ground state at** ν **= -2 with twisted angle** θ **= 1.21° under** U_d **= 0.07 eV displacement field and** B_z **= 2T**. without IVC order (**a**), with IVC order (**b**). The energy bands from K (K') valley are remarked with blue solid lines (red dashed lines) respectively. In the IVC state, all bands are present with green lines.

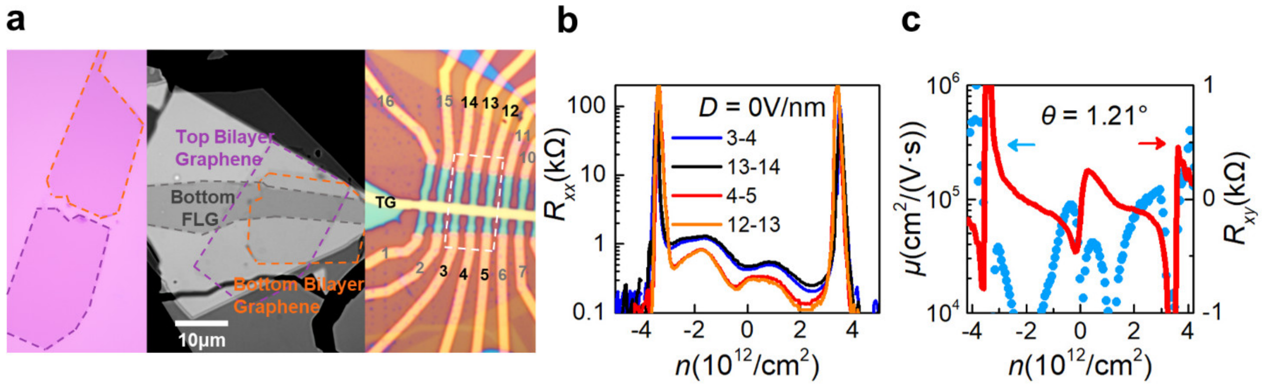

Supplementary Figure 5. **Device fabrication and homogeneity of twist angle of device D2**. **a**, Optical microscope images of the fabrication process. The bilayer graphene, ABBA-TDBG and the dual gate device are presented in turn from left to right. The scale bar is shown in figure. **b**, Four-terminal longitudinal resistance versus carrier density n at $D = 0$ V/nm between different bars. **c**, Hall mobility and Hall resistance versus carrier density n at $D = 0$ V/nm.

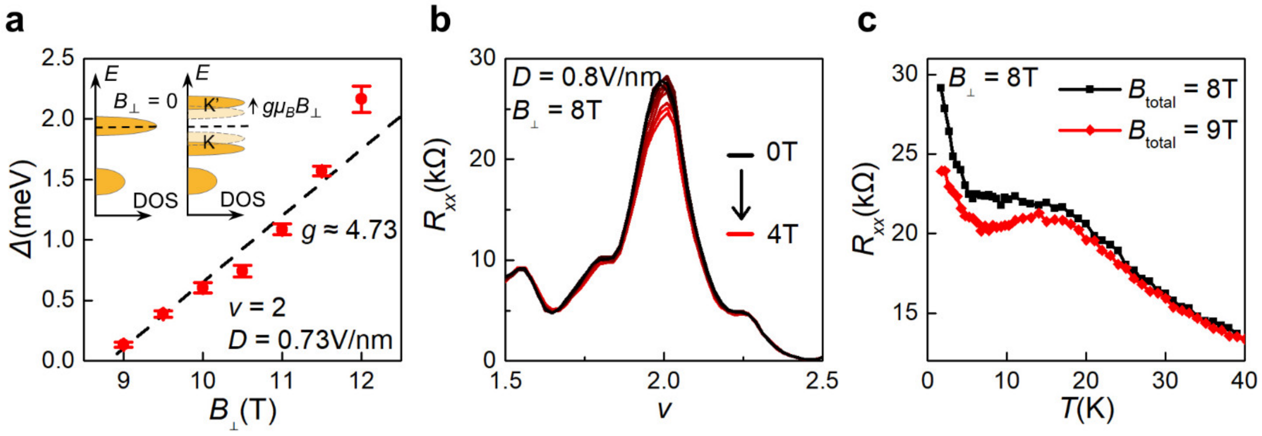

Supplementary Figure 6. **Competing between spin and valley polarization at $\nu = 2$ in device D2**. **a**, Thermal activation gaps versus perpendicular magnetic field. Inset, schematic of density of states at $B = 0$ T and finite perpendicular magnetic field. The valley degeneracy will be lifted with the orbital Zeeman effect. **b**, Longitudinal resistance R_{xx} versus filling factor ν under the tilt magnetic field. The perpendicular magnetic field is fixed at 8 T and the in-plane magnetic field increases from 0 to 4 T. **c**, Longitudinal resistance R_{xx} versus temperature T at $\nu = 2$ under the tilt magnetic field. The perpendicular magnetic field is fixed at 8 T and the total magnetic field increases from 8 to 9 T.

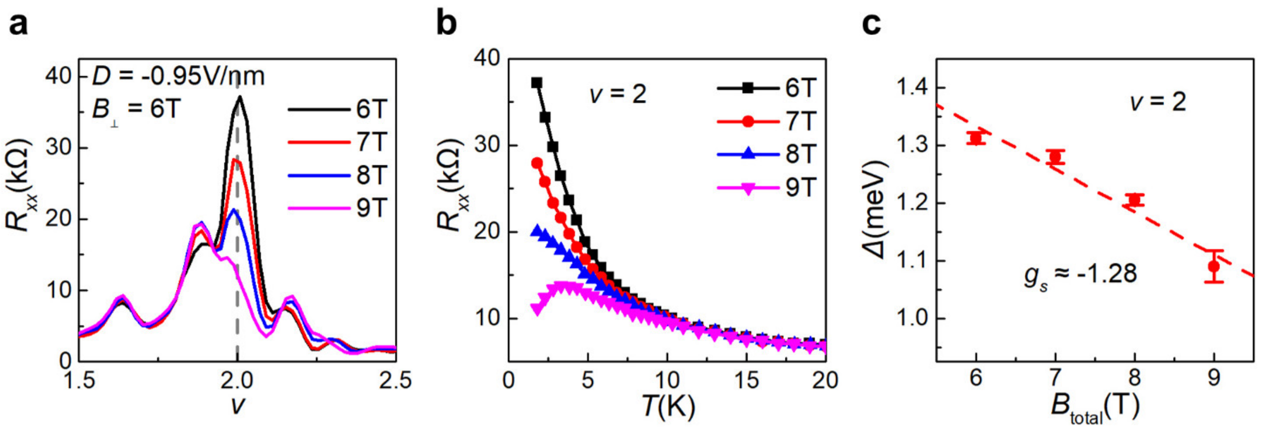

Supplementary Figure 7. **Competition between spin and valley polarization at $\nu = 2$ in device D1**. **a**, Longitudinal resistance R_{xx} versus filling factor ν under the tilted magnetic field. Here the perpendicular component is fixed at 6T and B_{total} increases from 6T to 9T. **b**, Longitudinal resistance R_{xx} versus Temperature T of $\nu = 2$ insulators shown in **a**. **c**, Thermal activation gaps versus B_{total} of $\nu = 2$ insulators.

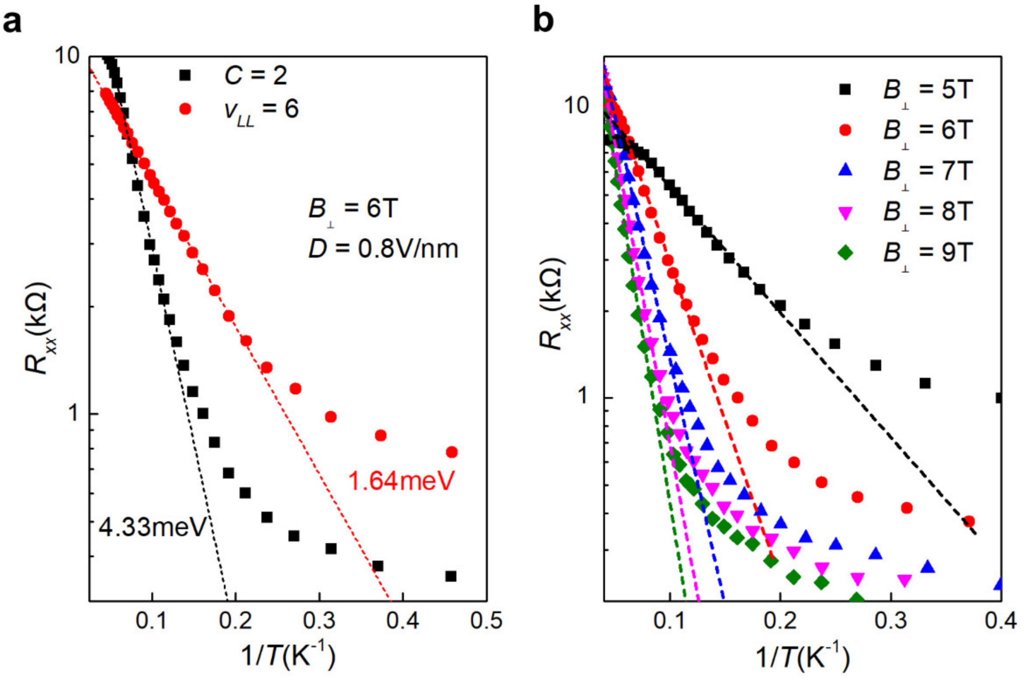

Supplementary Figure 8. **The energy gap of the $C = 2$ Chern insulator at $D = 0.8$ V/nm in Device D2**. **a**, Temperature dependence of R_{xx} at $B_{\perp} = 6$ T. The red dots and black dots correspond to the R_{xx} dip of $C = 2$ Chern insulator and the LL with $\nu_{LL} = 6$, respectively. **b**, Longitudinal R_{xx} of the Chern insulator versus Temperature T under the perpendicular magnetic field. The dashed lines in **a** and **b** are linear fittings according to the thermal activation behavior, $R_{xx} \sim \exp(-\Delta/2kT)$.

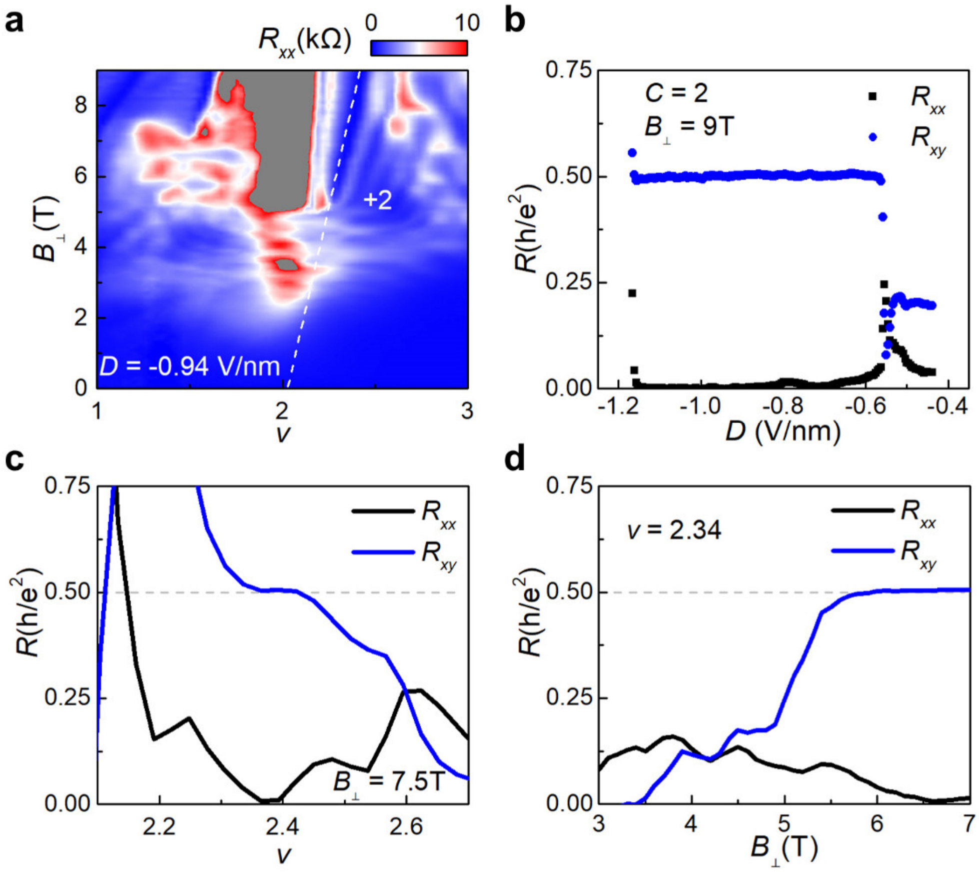

Supplementary Figure 9. ***C*** **= 2 Chern insulator in device D1**. **a**, Longitudinal resistance R_{xx} as a function of filling factor v and perpendicular magnetic field $B_{\perp}$. The white dash line corresponds to the $C = 2$ Chern insulator emanating from $v = 2$. **b**, Longitudinal resistance R_{xx} and Hall resistance R_{xy} of the $C = 2$ Chern insulator versus displacement field D at $B_{\perp} = 9$ T. **c**, Line cuts of R (v, $B_{\perp}$) at $B_{\perp} = 7.5$ T. (d) Line cuts of R (v, $B_{\perp}$) at $v = 2.34$.

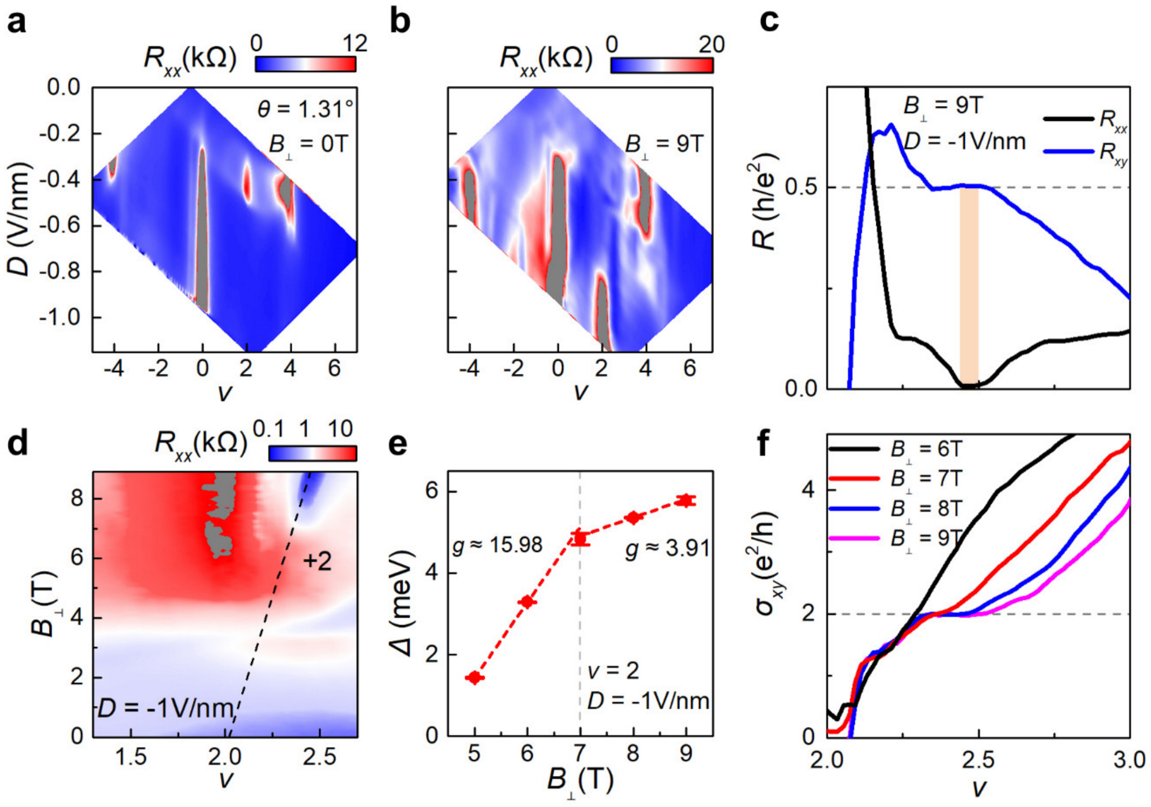

Supplementary Figure 10. **Valley polarization and the Chern insulator in device D3 ($\theta = 1.31°$)**. **a**, **b**, Longitudinal resistance R_{xx} as a function of filling factor v and displacement field D at $B_{\perp} = 0$ T and 9 T, respectively. The spin polarized correlated insulating state appears at $v = 2$ and $B_{\perp} = 0$ T. It disappears with the increase of $B_{\perp}$, while a valley polarized state accompanied with a Chern insulator develops at a larger electric field. **c**, Line cuts of R (v, $B_{\perp}$) at $B_{\perp} = 9$ T. It shows the quantized R_{xy} and minimum R_{xx} of the $C = 2$ Chern insulator. **d**, Longitudinal resistance R_{xx} as a function of filling factor v and perpendicular magnetic field $B_{\perp}$ at $D = -1$ V/nm shows the $C = 2$ Chern insulator emanating from $v = 2$. **e**, The thermal activation gap versus perpendicular magnetic field $B_{\perp}$ at $v = 2$. According to Zeeman effect, the effective g factor can be extracted by linear fitting. $g \approx 15.98$ from $B_{\perp} = 5$ T to 7 T and $g \approx 3.98$ from $B_{\perp} = 7$ T to 9 T indicate the g factor is magnetic field dependent in this device. Besides, the large g factor suggests the main contribution to the energy gap comes from the orbital Zeeman effect. **f**, Hall conductance σ_{xy} versus filling factor v under the perpendicular magnetic field.

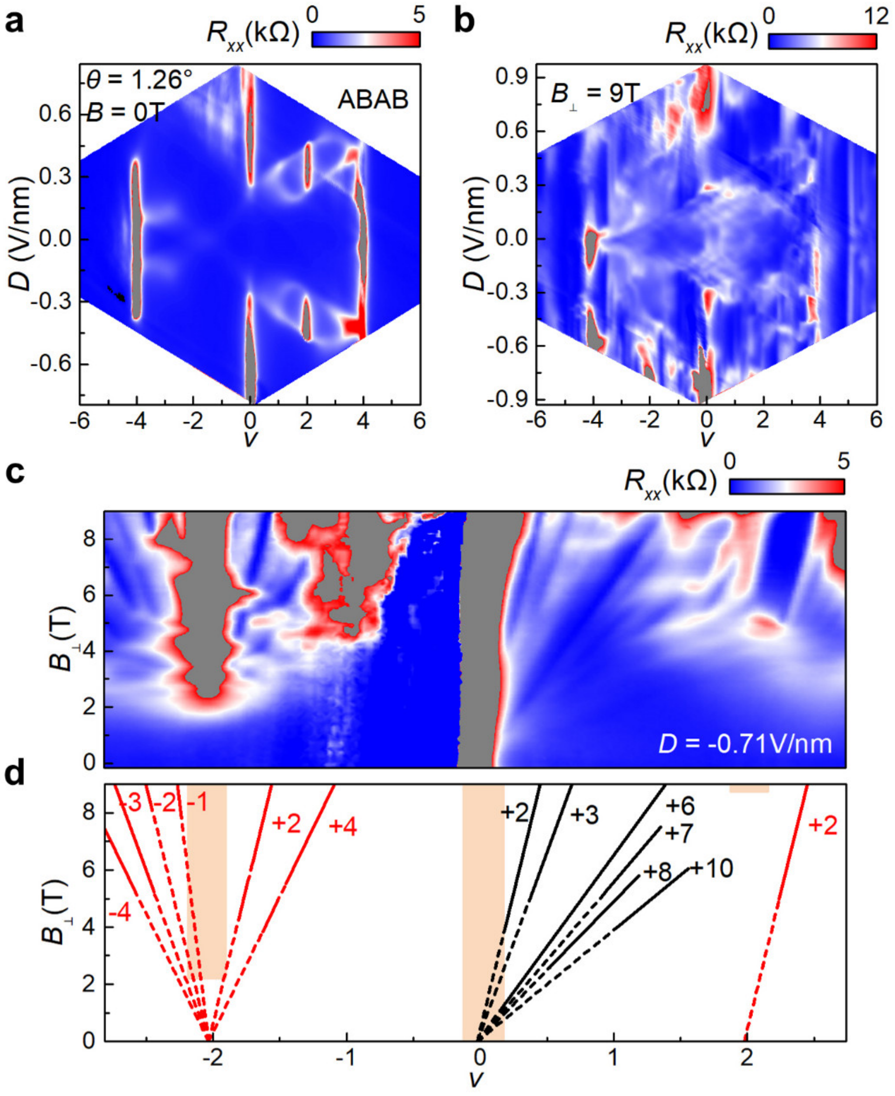

Supplementary Figure 11. **Valley polarized states in ABAB-stacked TDBG**. **a**, **b**, Longitudinal resistance R_{xx} as a function of filling factor ν and displacement field D at B = 0T and $B_\perp$ = 9T, respectively. **c**, Landau fan diagram at D = -0.71V/nm. **d**, schematic of Landau levels and the Chern insulator shown in **c**.

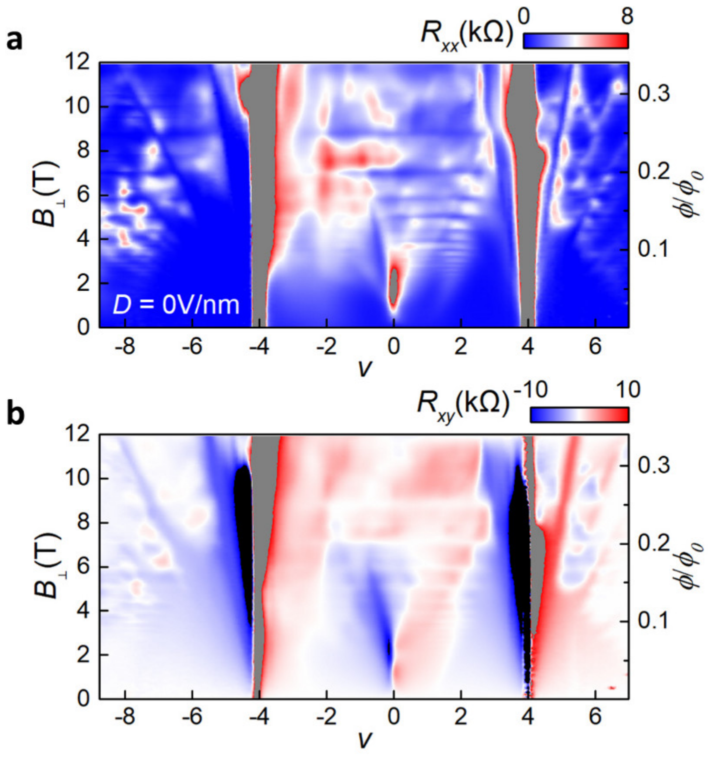

Supplementary Figure 12. **Landau fan diagram of device D2 at D = 0**. **a**, **b**, Longitudinal resistance R_{xx} and Hall resistance R_{xy} as a function of filling factor v and perpendicular magnetic field $B_\perp$. The periodic oscillation of resistance related to the magnetic flux per moiré unit cell is known as the Brown-Zak oscillation. The accurate twisted angle can be extracted from the oscillation period.

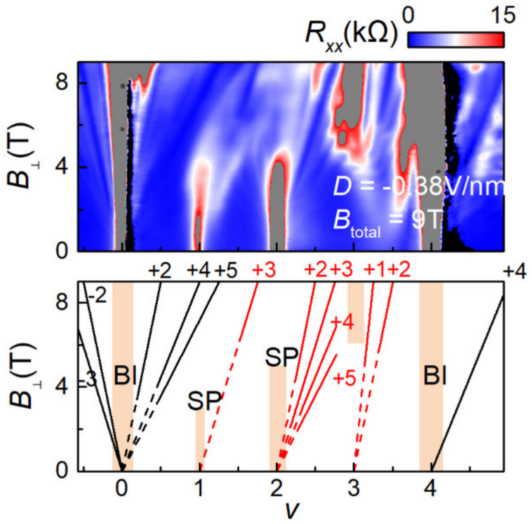

Supplementary Figure 13. **Landau fan diagram of device D2 at D = -0.38 V/nm under the tilt magnetic field**. Top, longitudinal resistance Rxx as a function of v and B⊥ at D = -0.38 V/nm. Here the magnetic field is fixed at 9 T and the direction is changed from in-plane to perpendicular. Bottom, schematic of LLs and insulating states shown in the top panel.

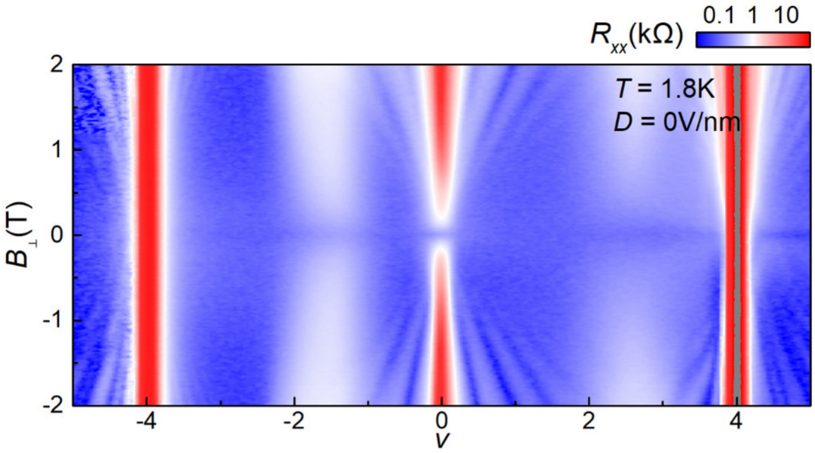

Supplementary Figure 14. **Landau fan diagram of device D1 at *D* = 0V/nm**.

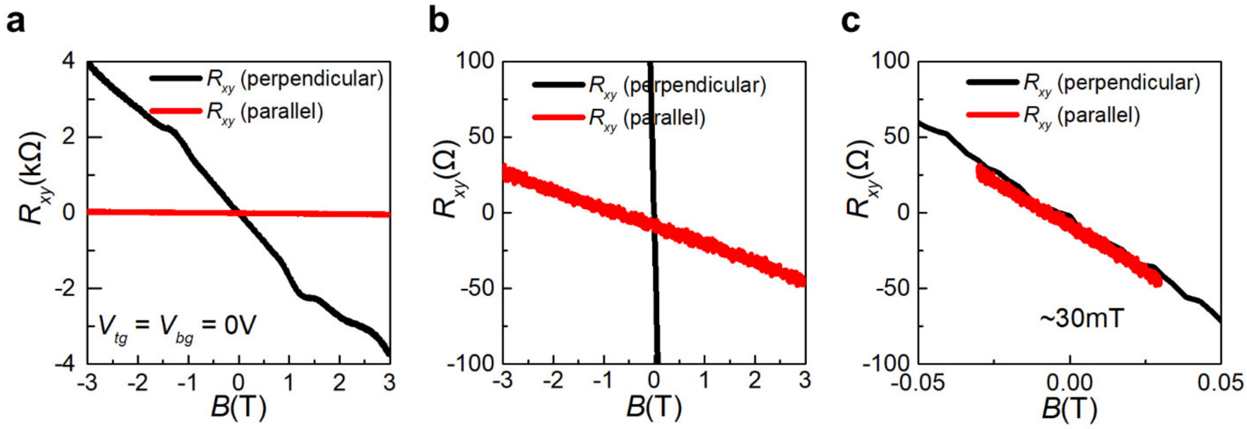

Supplementary Figure 15. **Calibration of the field direction**. **a-c** Hall resistance R_{xy} versus magnetic field B in perpendicular and parallel directions. In figure **c**, the parallel magnetic field are reduced by ~100 times to compare with the perpendicular magnetic field.

Supplementary References:

1. Liu, J., Ma, Z., Gao, J. & Dai, X. Quantum valley Hall effect, orbital magnetism, and anomalous Hall effect in twisted multilayer graphene systems. *Phys. Rev. X* **9**, 031021 (2019).
2. Koshino, M. Band structure and topological properties of twisted double bilayer graphene. *Phys. Rev. B* **99**, 235406 (2019).
3. Zhang, S., Dai, X. & Liu, J. Spin-Polarized Nematic Order, Quantum Valley Hall States, and Field-Tunable Topological Transitions in Twisted Multilayer Graphene Systems. *Phys. Rev. Lett.* **128**, 026403 (2022).
4. Moon, P. & Koshino, M. Optical absorption in twisted bilayer graphene. *Phys. Rev. B* **87**, 205404 (2013).
5. Bistritzer, R. & MacDonald, A. H. Moiré bands in twisted double-layer graphene. *Proc. Natl Acad. Sci. USA* **108**, 12233 (2011).
6. Koshino, M. *et al.* Maximally localized Wannier orbitals and the extended Hubbard model for twisted bilayer graphene. *Phys. Rev. X* **8**, 031087 (2018).
7. Liu, J., Liu, J. & Dai, X. Pseudo Landau level representation of twisted bilayer graphene: Band topology and implications on the correlated insulating phase. *Phys. Rev. B* **99**, 155415 (2019).
8. Song, Z.-D. *et al.* First principle calculation of the effective Zeeman's couplings in topological materials. Preprint at https://arxiv.org/abs/1512.05084v3 (2015).
9. Koshino, M. Chiral orbital current and anomalous magnetic moment in gapped graphene. *Phys. Rev. B* **84**, 125427 (2011).
10. Lee, J. Y. *et al.* Theory of correlated insulating behaviour and spin-triplet superconductivity in twisted double bilayer graphene. *Nat. Commun.* **10**, 5333 (2019).
11. Wu, Q., Liu, J., Guan, Y. & Yazyev, O. V. Landau levels as a probe for band topology in graphene moiré superlattices. *Phys. Rev. Lett.* **126**, 056401 (2021).
12. Sun, S., Song, Z., Weng, H. & Dai, X. Topological metals induced by the Zeeman effect. *Phys. Rev. B* **101**, 125118 (2020).
